# Perioperative Complications of Patients with SARS-CoV-2 Infection in Neurosurgery

**DOI:** 10.3390/jcm11030657

**Published:** 2022-01-27

**Authors:** Ladina Greuter, Christian Zweifel, Raphael Guzman, Jehuda Soleman

**Affiliations:** 1 Department of Neurosurgery, University Hospital of Basel, 4031 Basel, Switzerland; christian.zweifel@ksgr.ch (C.Z.); raphael.guzman@usb.ch (R.G.); jehuda.soleman@gmail.com (J.S.); 2 Department of Neurosurgery, Cantonal Hospital Graubünden, 7000 Chur, Switzerland; 3 Faculty of Medicine, University of Basel, 4056 Basel, Switzerland

**Keywords:** SARS-CoV-2, COVID-19, neurosurgery, complications

## Abstract

Background: The outbreak of coronavirus disease 2019 (COVID-19) has been rapidly evolving, resulting in a pandemic, with 270,031,622 infections according to the World Health Organization. Patients suffering from COVID-19 have also been described to suffer from neurologic and coagulopathic symptoms apart from the better-known flu-like symptoms. Some studies showed that patients suffering from COVID-19 were likely to developed intracranial hemorrhages. To our knowledge, only a few studies have investigated postoperative complications in COVID-19-positive neurosurgical patients and investigated the perioperative complications, either thrombotic or hemorrhagic, in patients with SARS-CoV-2 undergoing a neurosurgical intervention. Methods: We conducted a retrospective cohort study including patients from March 2020 to March 2021 undergoing neurosurgical interventions and suffering from COVID-19. Our primary outcome parameter was a hemorrhagic or thrombotic complication within 30 days after surgery. These outcomes were compared to those for a COVID-19-negative cohort of patients using propensity score matching. Results: We included ten COVID-19-positive patients with a mean age of 56.00 (±14.91) years. Twelve postoperative complications occurred in five patients. Three thrombotic complications (30%) were observed, with two cerebral sinus vein thromboses and one pulmonary embolus. Two patients suffered from a postoperative hemorrhagic complication (20%). The mean postoperative GCS was 14.30 (±1.57). COVID-19-positive patients showed a significantly higher rate of overall postoperative complications ((6 (60.0%) vs. 10 (19.2%), *p* = 0.021), thrombotic complications (3 (30.0%) vs. 1 (1.9%), *p* = 0.009), and mortality (2 (20.0%) vs. 0 (0.0%), *p* = 0.021) compared to the matched cohort of COVID-19-negative patients, treated at our institute before the SARS-CoV-2 pandemic. Conclusion: Patients undergoing neurosurgical operations with concomitant COVID-19 infection have higher rates of perioperative complications.

## 1. Introduction

The outbreak of the coronavirus disease (COVID-19), originating in Wuhan, China, at the end of 2019, was caused by the severe acute respiratory syndrome coronavirus 2 (SARS-CoV-2) [1]. According to the World Health Organization (WHO), it has been rapidly spreading all over the world, causing 270,031,622 infections and 5,310,502 deaths by December 2021 [2]. It typically manifests as fever, cough, diarrhea, and fatigue; however, patients suffering from COVID-19 have also been described to suffer from neurologic symptoms [3,4]. These symptoms can include encephalopathy, cerebrovascular disease including acute stroke, cognitive impairment, seizures, and dysgeusia [3,5]. A multicenter European study showed that patients suffering from COVID-19 also developed intracranial hemorrhage (ICH) with a typical distribution of subarachnoid blood over the cerebral hemispheres [6]. Otherwise, a hypercoagulable state was reported in patients with COVID-19 infections. Especially, patients with a severe disease course were shown to have an up-to-six-times-elevated level of D-dimers, indicating a highly hypercoagulable state, predisposing them to thrombotic events [7,8]. It is hypothesized that small and large cerebral vessels are affected by different mechanisms resulting in either ischemia or bleeding [9]. There are several reports of patients suffering from COVID-19 presenting with a cranial sinus venous thrombosis (CSVT) without suffering from any of the typical risk factors [10,11]. It has been shown that patients undergoing general surgery with a concomitant COVID-19 infection are prone to developing postoperative complications [12]. To our knowledge, several studies have focused on the changes in referrals and organization of neurosurgical units during the SARS-CoV-2 pandemic but only few studies have investigated the incidence of perioperative complications in patients undergoing cranial neurosurgical procedures with concomitant COVID-19 infection [13,14,15,16]. This study aimed to investigate the rate of perioperative hemorrhagic or thrombotic complications in SARS-CoV-2-positive patients undergoing neurosurgical procedures. 

## 2. Materials and Methods

We retrospectively analyzed consecutive patients ≥18 years of age undergoing cranial neurosurgical procedures with a concomitant COVID-19 infection, between March 2020 and March 2021, at our institution. All the electively scheduled patients received a SARS-CoV-2 test before or at hospital admission, and if medically possible, positively tested patients were rescheduled for surgery after a ten-day quarantine period. Emergent admissions received a SARS-CoV-2 test at the first time point possible after emergent surgery. Quantitative SARS-CoV-2 tests to measure the viral load were only standardly carried out for patients in the intensive care unit (ICU). Only patients already positively tested for COVID-19 at the time of surgery or within ten days after surgery were included. Data were collected from our surgical logbooks and patients’ medical files. Baseline characteristics including age, gender, the side and anatomic location of pathology, the volume of the lesion (calculated as AxBxC/2), the preoperative midline shift (MLS), the type of surgery, the preoperative Glasgow coma scale (GCS), the American Society of Anesthesiologist (ASA) score, comorbidities, the intake of blood thinners, the type of blood thinners (aspirin (ASA), clopidogrel, vitamin K antagonists (VKAs), a type of direct oral anticoagulant (DOAC), or heparin), and steroid application were collected and analyzed. To assess the individual patient’s risk for developing venous thromboembolism (VTE), we calculated the IMPROVE risk score for all the patients [17]. This score predicts the 3-month risk for developing a VTE in hospitalized patients by taking into account the patients’ mobility, duration of ICU admission, previous history of VTE or cancer, and age [17]. Additionally, we assessed the time spent in the intensive care unit, intubation and the duration of the intubation, oxygen treatment due to COVID-19 infection, antiviral therapy, and prophylactic heparin administration. The primary outcome measure was perioperative complications, either hemorrhagic or thrombotic, within 30 days after surgery. The secondary outcome measures were the postoperative GCS, postoperative modified Rankin Scale (mRS), 30-day mortality, overall mortality, morbidity, and revision surgery. The mean follow-up time was 102.29 (±59.33) days, with an outcome assessed for every patient. A favorable outcome was defined as mRS < 3. 

### Matching with a COVID-19-Negative Cohort

We compared the primary outcome as well as overall complications and mortality to those for a retrospective cohort of patients undergoing cranial surgery at our institution from 2009 until 2019. To provide a better comparison between the control group and the COVID-19 group, the groups were matched for gender, ASA score, pathology type, and location of pathology. The matching was carried out based solely on clinical and not numerical (e.g., 1:1) matching. 

Descriptive statistics were completed for all the parameters. For numerical values, the chi square test was used, while for continuous variables, Student’s *t*-test was applied. All the statistical analyses were carried out using the R statistical software (R Foundation for Statistical Computing, Vienna, Austria, version 3.6.2, 2019). A value of *p* < 0.05 was considered significant. The study was approved by the local ethics committee (EKNZ, Basel, Switzerland, Project ID 2021-00218), who waived patients’ consent. 

## 3. Results

Out of 494 screened patients, undergoing cranial neurosurgical procedures from March 2020 to March 2021, ten patients tested positive for COVID-19 and were included in this study. The mean age was 56.00 (±14.91) years (20% females). Five patients (50%) underwent elective procedures, while the other half underwent emergent surgery. Four patients (40%) presented with a brain tumor (epidermoid, meningioma, metastasis of a pulmonary adenocarcinoma, and pituitary adenoma), two patients (20%) suffered from a postoperative infection, one patient (10%) suffered a traumatic brain injury (TBI) with an epidural bleed, and two patients (20%) were treated for an aneurysm, of which one presented with an acute SAH (Table 1). Two patients (20%) were found to be SARS-CoV-2 positive before surgery (diagnosed 15 and 4 days before surgery); the remaining eight patients (80%) tested positive after undergoing surgery (mean, 5.75 (±3.96) days after surgery). One of the two patients testing positive before surgery showed no symptoms due to COVID-19, after having suffered a previous symptomatic COVID-19 infection several weeks earlier. The other patient with a preoperative positive SARS-CoV-2 test presented with meningitis and wound infection after microvascular decompression for hemifacial spasm and received emergent revision surgery and antibiotic therapy. A detailed overview of the patients’ demographics and clinical characteristics is shown in Table 1 and Table 2. 

### 3.1. Postoperative Complications

Twelve postoperative complications occurred in five patients (50%). Three postoperative thrombotic complications (30%) were observed, with two cerebral sinus vein thromboses and one pulmonary embolus. An additional patient had already presented with a jugular vein thrombosis at admission, which he developed before testing positive for SARS-CoV-2. All the patients received weight-adjusted low-dose heparin as prophylaxis on the first postoperative day. Patients with a thrombotic complication were treated with therapeutic heparin, which was converted to DOAC treatment for at least three months. Two patients suffered from a postoperative hemorrhagic complication (20%); one had a rebleed from a cerebellar metastasis and underwent emergent revision surgery, while the other patient had a fatal intraparenchymal hemorrhage two days after elective ventriculoperitoneal shunt surgery for postoperative hydrocephalus (Table 3). The latter received therapeutic heparin for treating a pulmonary embolism (PE) preoperatively. The other postoperative complications were surgical-site infection (SSI) (n = 1), hydrocephalus (n = 2), cerebrovascular vasospasm after subarachnoid hemorrhage (SAH) (n = 1), cerebral salt wasting syndrome (CSW) after SAH (n = 1), sepsis (n = 1), and prolonged elevated intracranial pressure (ICP) after TBI leading to a decompressive hemicraniectomy (n = 1). Four patients (40%) underwent a total of six additional surgeries within 30 days of the initial surgery (Table 1 and Table 3). 

### 3.2. Clinical Outcome and Mortality

The mean postoperative GCS and mRS were 14.30 (±1.57) and 1.50 (±1.58), respectively (Table 3). At follow up, all the patients presented with a GCS of 15 (±0) and a mean mRS of 0.62 (±1.41). Two out of ten patients (20%) died during their hospital stay. One patient died after 37 days due to a fatal intraparenchymal hemorrhage (IPH) two days after shunt insertion, while the other patient died of a septic shock and acute kidney failure 25 days after emergent empyema evacuation (Table 1 and Table 3). None of the patients died due to respiratory failure related to their COVID-19 infections. 

### 3.3. COVID-19 Symptoms and Treatment

Five patients (50%) showed no COVID-19-related symptoms during the hospital stay. Three patients (30%) presented only with fever, while two patients (20%) showed symptoms of rhinitis and dysgeusia in addition to fever. The mean postoperative time in the ICU was 4.70 (±7.36, range, 0–20) days. On average, patients remained intubated for 2.22 (±4.02, range, 0–11) days. Only one patient (10%) remained intubated due to COVID-19. Three patients (30%) received antiviral therapy with remdesivir for four days, while seven patients (70%) received oral steroids for a mean duration of 5.20 (±2.95) days. The mean overall length of stay was 23.30 (±18.28) days (Table 3). We did not correlate the complications with the severity of the SARS-CoV-2 infection, however, only one patient in our cohort required intubation due to COVID-19, while the others only suffered from milder symptoms. 

### 3.4. Comparison to a Matched COVID-19-Negative Cohort

COVID-19-positive patients showed a significantly higher rate of overall postoperative (6 (60.0%) and 10 (19.2%) patients, *p* = 0.021) and thrombotic complications (3 (30.0%) and 1 (1.9%) patient/s, *p* = 0.009), as well as mortality (2 (20.0%) and 0 (0.0%) patients, *p* = 0.021) compared to a matched historical cohort of COVID-19-negative patients, treated at our institute before the SARS-CoV-2 pandemic (Table 4). However, no statistically significant difference between the hemorrhagic complication rate of the COVID-19-positive patients and the control group was seen (2 (20.0%) and. 9 (17.3%) patients, *p* = 1).

## 4. Discussion

We present and analyze a series of ten patients undergoing neurosurgical procedures, while suffering from a concomitant COVID-19 infection. To our knowledge, this is one of a few case series specifically focusing on COVID-19-positive patients undergoing neurosurgical operations [12,13]. In this series, we observed thrombotic and hemorrhagic complication rates of 30% and 20%, respectively; however, none of these studies focused on neurosurgical patients [12]. Nepogodiev et al. showed that half of all patients undergoing surgery while suffering from COVID-19 developed pulmonary complications and had an increased mortality rate [12]. Several other studies report an association of intracranial hemorrhage in COVID-19-positive patients; however, the detailed pathophysiological mechanism and risk factors are not completely understood [6,9,18,19,20,21]. Mostly, peripheral subarachnoid hemorrhages (SAHs) over the convexities were observed in COVID-19-positive patients, while some studies also describe the development of IPH [6,9,19]. Three studies showed an endothelial vasculitis in several organs of COVID-19 patients, which might the cause for intracranial hemorrhages [9,21,22]. In imaging studies, an enhancement of middle and large cell intracerebral arteries was observed, indicating an inflammation process in the vessels [9,23]. Intracranial hemorrhages including SAH and IPH caused by different forms of vasculitis have been described in the past [24]. A similar pathophysiological process for intracranial hemorrhages in SARS-CoV-2 patients might be possible. Moreover, IPH can also be caused by CSVT, and especially in COVID-19-positive patients, who are known to be in a hypercoagulable state, some of the IPH observed could also be caused by CSVT [10]. In our case series, we observed two intraparenchymal hemorrhages but none of the patients presented with a SARS-CoV-2 -typical bleeding pattern. Whether the hemorrhages observed in our series were caused by SARS-CoV-2 or due to the underlying disease or neurosurgical procedure remains unknown. The COVID-19 group of our cohort did not show a significantly higher bleeding rate compared to the non-COVID-19 group. The patient with a fatal IPH after VP-shunt insertion was preoperatively anticoagulated with heparin due to a PE, which could have also increased the patient’s risk for a postoperative hemorrhage [25,26], although heparin was discontinued during the perioperative phase. Especially in COVID-19-positive patients, who are often treated with blood thinners, distinguishing between the different bleeding etiologies can be challenging [20,27]. In COVID-19, patients were shown to enter a hypercoagulable state with a higher rate of heparin-binding proteins, resulting in an elevated rate of thrombotic complications despite treatment with low-dose heparin for deep-vein thrombosis (DVT) prophylaxis [28,29,30]. A thrombosis rate of up to 20–30% was described in severely ill COVID-19 patients, with a stroke rate of up to 2% [30,31]. This rate is comparable to the rate of thrombotic complications in our cohort of COVID-19 patients (30%). One patient suffered from a PE, while two patients developed a postoperative CSVT, which is otherwise a rare finding after neurosurgical procedures [32,33]. Especially in neurosurgical patients, the treatment of CSVT can be challenging, as the risks of bleeding and a thromboembolic event need to be balanced [34]. In our cohort, both patients received a prophylactic dose of heparin (10,000 units/24 h) after diagnosis (4 days and 20 days after surgery, respectively), which was subsequently increased over the following 2–3 days until a therapeutic anti-Xa level of heparin was reached. Before the diagnosis of CSVT, both patients received the standard dose of prophylactic weight-adapted low-dose heparin, which might have been too low to adequately prevent CSVT in these COVID-19-positive patients [28]. It remains unclear whether patients with a COVID-19 infection undergoing a neurosurgical procedure should postoperatively receive a higher dose of heparin than usual, since the bleeding risk due to an elevated dose of heparin is expected to be minimal [35]. Moreover, certain neurosurgical pathologies such as meningioma, cerebral metastasis, or malignant brain tumors, such as glioblastoma, carry a high risk for thrombotic complications, independent from a COVID-19 infection. To better assess the individual patients’ risk for developing a thrombotic complication, we calculated the IMPROVE risk scores for all the patients. Since D-dimer levels were not routinely measured during the hospitalization, we calculated the IMPROVE and not IMPROVE-DD score [17,36,37]. However, we did not observe a clear association between a high IMPROVE score and the thromboembolic complication rates in our cohort. Based on our findings, the rate of thrombotic complications after neurosurgical procedures in patients with concomitant COVID-19 infection was nearly twice as high as that in the control group (3 (30.0%) vs. 1 (1.9%), *p* = 0.009, Table 4). Therefore, in patients undergoing cranial procedures, with a high thromboembolic risk profile and a concomitant COVID-19 infection, an intra-/perioperative and maybe higher postoperative (e.g., subtherapeutic) thrombosis prophylaxis seems justified. The baseline D-dimer levels in postoperative COVID-19-positive patients could be obtained in a prospective study to better assess the thromboembolic risk profile and complication rate in these patients. However, in COVID-19 patients undergoing neurosurgical procedures, the perfect balance between treating the procoagulatory state and the risk of postoperative rebleeding is difficult and yet to be defined. 

### Limitations

This is a retrospective case series and is subject to all the limitations inherent in such a study design. The statistical analyses carry a certain bias due to the low number of patients included. We tried to minimize this bias by matching the groups for certain baseline characteristics, through propensity score matching. The low number of included patients might be due to the fact that, in patients with a known COVID-19 infection, surgery is usually postponed, if possible. Hence, in most of the patients, COVID-19 was diagnosed after surgery, or they were in need of emergency care. Additionally, patients testing positive for SARS-CoV-2 after their hospital discharge might have been missed, as their test result, if produced at a different institution, is not available in our electronic patient records. Moreover, this study was carried out before vaccination was available in Switzerland. Therefore, no conclusions regarding vaccinated patients can be drawn. 

## 5. Conclusions

Patients undergoing neurosurgical procedures with concomitant COVID-19 infection have a higher postoperative thrombotic complication rate. However, the postoperative outcomes were favorable in most of the included patients, and therefore, if indicated, neurosurgical procedures should not be postponed due to COVID-19. 

## Figures and Tables

**Table 1:** Overview of patients. Abbreviations: No = number, CPA = cerebellopontine angle, SAH = subarachnoid hemorrhage, VA = vertebral artery, GBM = glioblastoma, MCA = middle cerebral artery, EVD = external ventricular drain, ETV = endoscopic third ventriculostomy, PE = pulmonary emboli, SSI = surgical site infection, IPH = intraparenchymal hemorrhage, CSVT = cerebral sinus vein thrombosis, ICP = intracranial pressure, VP shunt = ventriculoperitoneal shunt, CSW = cerebral salt wasting syndrome.

Patient No	Age	Gender	Pathology	1st Surgery	Time of COVID-19 Infection (Days) *	Antiviral Therapy	ICU (Days)	Intubation (Days)	IMPROVE Risk Score	Thromboembolic Complications	Hemorrhagic Complications	Other Complications	30-Day Revision Surgery	Mortality (Cause of Death)
**1**	61	M	CPA Epidermoid	Retrosigmoid Craniotomy	6	yes	8	7	7	PE	IPH	SSI, hydrocephalus	Wound revision, VP shunt	yes (IPH)
**2**	61	M	SAH (mFisher 4) and VA aneurysm	EVD, stent-assisted Coiling	10	none	16	0	3	-	-	Vasospasm, CSW, hydrocephalus	ETV and VP shunt	no
**3**	66	M	Subdural empyema after resection of a GBM	Temporal craniectomy	8	none	0	0	3	-	-	Sepsis	-	yes (sepsis)
**4**	74	F	Cerebellar metastasis of a pulmonary adenocarcinoma	Paramedian suboccipital craniotomy	10	none	7	2	4	CSVT	IPH	-	Re-craniotomy	no
**5**	70	M	SSI and meningitis after facial hemispasm with neurovascular conflict	Retrosigmoid craniotomy as revision	- 4	yes	0	0	4	-	-	-	-	no
**6**	60	F	MCA aneurysm	Pterional craniotomy and clipping	7	none	1	0	1	-	-	-	-	no
**7**	24	M	Pituitary adenoma and acromegaly	Endoscopic transsphenoidal surgery	1	none	0	0	0	-	-	-	-	no
**8**	48	M	Epidural hematoma	Frontotemporal craniotomy	0	yes	20	11	2	CSVT	-	ICP crisis	Hemicraniectomy	no
**9**	41	M	Falx meningioma	Frontal craniotomy	5	none	1	0	6	-	-	-	-	no
**10**	55	M	Falx meningioma	Frontal craniotomy	0 ^§^	none	1	0	3	-	-	-	-	no

* in relation to primary surgery. ^§^ patient remained SARS-CoV-2 positive at admission after having suffered from COVID-19 several weeks before surgery.

**Table 2:** Demographic data. Abbreviations: SD = standard deviation, ASA Score = American Society of Anesthesiologist Score, GCS = Glasgow Coma Scale, MLS = midline shift, EVD = external ventricular drain, DM = diabetes mellitus, CAD = coronary artery disease, CVD = cardiovalvular disease, COPD = chronic obstructive pulmonary disease, CKD = chronic kidney disease, VTE = venous thromboembolism.

	Overall
n	10
**Age (mean (±SD)), years**	56.00 (±14.91)
**Gender = Male (%)**	8 (80.0)
**ASA Score (mean (±SD))**	3.20 (±0.79)
**GCS preoperative (mean (±SD))**	13.60 (±2.95)
**Pathology (%)**	
Tumor	5 (50.0)
Neurotrauma/Infection	2 (20.0)
Vascular pathology	3 (30.0)
**Size of pathology (mean (±SD)), mm** [3]	218.33 (±287.39)
**MLS (mean (±SD)), mm**	2.30 (±4.11)
**Surgery (%)**	
Craniotomy	8 (80.0)
Endoscopic transsphenoidal surgery	1 (10.0)
EVD	1 (10.0)
**Comorbidities (%)**	
Hypertension = yes (%)	5 (50.0)
DM = yes (%)	1 (10.0)
CAD = yes (%)	2 (20.0)
CVD = yes (%)	1 (10.0)
COPD = yes (%)	1 (10.0)
CKD = yes (%)	0 (0.0)
Smoking = yes (%)	4 (40.0)
Alcohol = yes (%)	3 (30.0)
Thromboembolic events = yes (%)	3 (30.0)
IMPROVE Risk Score VTE (±SD)	3.30 (±2.11)
**Blood Thinners = Yes (%)**	6 (60.0)
**Types of Blood thinners (%)**	
Low-dose aspirin	4 (66.7)
Heparin	1 (16.7)
Rivaroxaban	1 (16.7)
**Preoperative Neurologic Symptoms (%)**	
Cranial nerve deficit	3 (30.0)
Acromegaly	1 (10.0)
Confusion	2 (20.0)
Hemiparesis	2 (20.0)
None	2 (20.0)
**COVID-19 Symptoms (%)**	
Fever	3 (30.0)
Rhinitis	2 (20.0)
None	6 (60.0)

**Table 3:** Postoperative clinical outcomes. Abbreviations: GCS = Glasgow Coma Scale, mRS = modified Rankin Scale, SD = standard deviation, ICU = intensive care unit, LOS = length of stay, FU = follow up.

	Overall
n (%)	10
**GCS postoperative (mean (±SD))**	14.30 (±1.57)
**mRS postoperative (mean (±SD))**	1.50 (1.58)
**Postoperative favorable outcome (mRS < 3) = yes (%)**	8 (80)
**Clinical improvement postoperative = better (%)**	6 (60.0)
**Postoperative ICU = yes (%)**	7 (70.0)
**Days ICU (mean (±SD))**	4.70 (±7.36)
**Postoperative intubation = yes (%)**	3 (30.0)
**Intubation due to COVID-19 = yes (%)**	1 (10.0)
**Intubation days (mean (±SD))**	2.22 (±4.02)
**Postoperative complication = yes (%)**	5 (50.0)
**Thromboembolic complication = yes (%)**	3 (30.0)
**Bleeding complication = yes (%)**	2 (20.0)
**Revision surgery = yes (%)**	6 (60.0)
**LOS (mean (±SD))**	23.30 (±18.28)
**Discharge location (%)**	
Home	5 (50)
Hospice	1 (10)
Rehabilitation	2 (20)
**In-hospital mortality = yes (%)**	2 (20)
**GCS at FU (mean (±SD))**	15.00 (±0.00)
**mRS at FU (mean (±SD))**	0.20 (±0.45)
**Favorable outcome at FU (mRS < 3) = yes (%)**	5 (50)

**Table 4:** Comparison of COVID-19-positive with negative patients undergoing neurosurgical operations at our institution regarding their outcomes.

Matched Cohort			
COVID-19	Negative	Positive	*p*-Value
n (%)	52	10	
**Age (mean (±SD))**	63.50 (±13.39)	56.00 (±14.91)	0.784
**Gender (male %)**	28 (56.0)	8 (80.0)	0.289
**Hypertension = yes (%)**	29 (55.8)	5 (50.0)	1.0
**Diabetes Mellitus Type 2 = yes (%)**	3 (5.8)	1 (10.0)	1.0
**Coronary Artery Disease = yes (%)**	10 (19.2)	2 (20.0)	1.0
**ASA Score (±SD)**	3.12 (±0.43)	3.20 (±0.79)	0.625
**Pathology (%)**			0.135
**Aneurysm**	20 (38.5)	2 (20.0)	
**Glioma**	15 (28.8)	1 (10.0)	
**Meningioma**	9 (17.3)	2 (20.0)	
**Metastasis**	1 (1.9)	1 (10.0)	
**Other**	7 (13.5)	4 (40.0)	
**Postoperative Complication = yes (%)**	10 (19.2)	6 (60.0)	**0.021**
**Thrombotic Complication = yes (%)**	1 (1.9)	3 (30.0)	**0.009**
**Bleeding Complication = yes (%)**	9 (17.3)	2 (20.0)	1
**In-Hospital Mortality = yes (%)**	0 (0.0)	2 (20.0)	**0.021**

## Data Availability

The data presented in this study are available on request from the corresponding author. The data are not publicly available due to ethical and privacy concerns.
